# Pulmonary, ocular and cutaneous Kaposi sarcoma in a HIV-infected patient

**DOI:** 10.1186/1471-2334-14-S7-P95

**Published:** 2014-10-15

**Authors:** Mihaela Ionică, Șerban Benea, Alina Cozma, Lia Cavaropol, Elisabeta Benea

**Affiliations:** 1 National Institute for Infectious Diseases "Prof. Dr. Matei Balş", Bucharest, Romania

## Background

Pulmonary Kaposi sarcoma involvement is frequent in HIV-infected patients, and the diagnosis is more difficult in severe immunosuppressed. On the other hand, HIV-infected patients tend to develop malignant tumors, such as Kaposi sarcoma. Although cutaneous involvement is the most common manifestation, extracutaneous involvement is also possible.

## Case report

We present the case of a 33 year-old male patient, HIV infected, with virological failure, who also had cutaneous (proved by histopathological exam of a cutaneous specimen) and ocular Kaposi sarcoma, prolonged fever and some pulmonary symptoms: cough, dyspnea, hypoxemia. Using bronchoscopy and pulmonary CT scan, we were able to diagnose the pulmonary involvement of Kaposi sarcoma.

## Conclusion

Initial antibiotic and later antifungal therapy did not improve the pulmonary symptomatology (chest X-ray revealed bilateral bronchopneumonia); the evolution was slowly favorable only after the optimal control of HIV infection by antiretroviral therapy.

## Consent

Written informed consent was obtained from the patient for publication of this Case report and any accompanying images. A copy of the written consent is available for review by the Editor of this journal.

